# Retraction: Khan et al. Structural and Magnetic Response in Bimetallic Core/Shell Magnetic Nanoparticles. *Nanomaterials* 2016, *6*, 72

**DOI:** 10.3390/nano7040073

**Published:** 2017-03-28

**Authors:** Nanomaterials Editorial Office

**Affiliations:** MDPI AG, St. Alban-Anlage 66, 4052 Basel, Switzerland; nanomaterials@mdpi.com

It has come to our attention that Figure 3 of the title paper [1] contains unacceptable levels of image manipulation and thus does not provide firm evidence of the particles reported. In particular, particles have been copied and pasted to make multiple copies in a single image, and the high contrast between the inner and outer shells of the particles are not plausible given their supposed composition. Further images provided by the authors exhibited the same flaws in contrast. As such, the conclusions of the paper are no longer supported and the paper will be marked as retracted.

We apologize to readers of *Nanomaterials* that this issue was not detected earlier and wish to thank the reader who brought this issue to our attention. *Nanomaterials* is a member of the Committee on Publication Ethics (COPE) and strives to uphold the highest ethical standards; extensive manipulation of images is not acceptable and we are committed to taking appropriate action when such cases are reported.
